# The cell surface hyaluronidase TMEM2 regulates cell adhesion and migration *via* degradation of hyaluronan at focal adhesion sites

**DOI:** 10.1016/j.jbc.2021.100481

**Published:** 2021-02-26

**Authors:** Fumitoshi Irie, Yuki Tobisawa, Ayako Murao, Hayato Yamamoto, Chikara Ohyama, Yu Yamaguchi

**Affiliations:** 1 Human Genetics Program, Sanford Burnham Prebys Medical Discovery Institute, La Jolla, California, USA; 2 Department of Urology, Hirosaki University Graduate School of Medicine, Hirosaki, Japan

**Keywords:** TMEM2, hyaluronan, hyaluronidase, extracellular matrix, focal adhesion, cell adhesion, cell migration, integrin, cDNA, complementary DNA, DTSSP, 3',3'-dithiobis(sulfosuccinimidyl propionate), ECD, extracellular domain, ECM, extracellular matrix, FA, focal adhesion, FA-HA, fluorescein-labeled HA, FBS, fetal bovine serum, HA, hyaluronan, HBSS++, Hank's balanced salt solution containing calcium and magnesium, HEK293, human embryonic kidney 293 cells, TMEM2, transmembrane protein 2, WGA, wheat germ agglutinin

## Abstract

The extracellular matrix (ECM) plays an important role in maintaining tissue homeostasis and poses a significant physical barrier to *in vivo* cell migration. Accordingly, as a means of enhancing tissue invasion, tumor cells use matrix metalloproteinases to degrade ECM proteins. However, the *in vivo* ECM is comprised not only of proteins but also of a variety of nonprotein components. Hyaluronan (HA), one of the most abundant nonprotein components of the interstitial ECM, forms a gel-like antiadhesive barrier that is impenetrable to particulate matter and cells. Mechanisms by which tumor cells penetrate the HA barrier have not been addressed. Here, we demonstrate that transmembrane protein 2 (TMEM2), the only known transmembrane hyaluronidase, is the predominant mediator of contact-dependent HA degradation and subsequent integrin-mediated cell–substrate adhesion. We show that a variety of tumor cells are able to eliminate substrate-bound HA in a tightly localized pattern corresponding to the distribution of focal adhesions (FAs) and stress fibers. This FA-targeted HA degradation is mediated by TMEM2, which itself is localized at site of FAs. TMEM2 depletion inhibits the ability of tumor cells to attach and migrate in an HA-rich environment. Importantly, TMEM2 directly binds at least two integrins *via* interaction between extracellular domains. Our findings demonstrate a critical role for TMEM2-mediated HA degradation in the adhesion and migration of cells on HA-rich ECM substrates and provide novel insight into the early phase of FA formation.

Hyaluronan (HA) is a high–molecular weight polysaccharide belonging to the family of glycosaminoglycans. It is a long unbranched polymer composed of repeating disaccharide units of a *N*-acetylglucosamine and a glucuronic acid, with a molecular weight reaching as high as 10^7^ Da (1). Because of its highly hydrophilic nature, HA has an extremely large hydrodynamic volume in solution and assumes semiflexible secondary and tertiary conformations based on intramolecular and intermolecular hydrogen bonding (2, 3). Furthermore, HA associates with a variety of matrix proteins and proteoglycans, such as aggrecan-type chondroitin sulfate proteoglycans and link proteins (4), to form higher-order complexes of extracellular matrix (ECM) molecules. Because of these unique properties, HA exerts a profound influence on the biomechanical properties of the ECM. Most notably, HA forms a gel-like meshwork that is permeable to small molecules but impermeable to particulate matter, including cells (5, 6, 7). While HA coated on a glass or plastic substrate can mediate weak attachment of cells that express HA receptors, such as CD44, high levels of HA in the extracellular and pericellular space act as a repulsive barrier to cell adhesion and migration. This antiadhesive effect is primarily because of steric exclusion by the thick, impermeable, and gel-like HA matrix, which is impenetrable to particles and cells, preventing the engagement of cell surface adhesion receptors with their ECM ligands (8, 9, 10).

HA is a major factor in defining the biophysical and biological properties of the tumor microenvironment. HA accumulation is especially prominent in tumors that exhibit desmoplastic reactivity (11, 12). It has been shown that HA deposited in the stroma of adenocarcinomas is produced predominantly by stromal cells, rather than by neoplastic tumor cells (13, 14, 15, 16), whereas hyaluronidase activities are associated with the neoplastic cells (17). Based on the antiadhesive properties of HA, it is thus reasonable to envision that tumor cells utilize degradation of extracellular HA as a means of enhancing cell–matrix adhesion and generating a milieu that favors tumor survival, growth, and invasion. The hyaluronidase activity of tumor cells is therefore complementary to their robust matrix metalloproteinase-based ability to degrade protein components of the ECM as an additional means of remodeling the microenvironment in a way that is favorable for tumor growth and invasion.

Since the 1990s, studies on HA degradation have mostly focused on the HYAL family proteins (*e.g.*, HYAL1, HYAL2) (13, 18). Although there are some indications that HYAL family proteins can be present on the cell surface (19, 20), multiple pieces of evidence indicate that they are primarily localized intracellularly and function in the context of lysosomes and endosomes (21, 22, 23). The distinctly acidic pH optimum for HYAL hyaluronidase activity (22, 24) (*e.g.*, pH 4 for HYAL2) is also consistent with that expected of lysosomal enzymes. In seeking to identify a hyaluronidase that physiologically functions on the cell surface, we have succeeded in demonstrating that the transmembrane protein 2 (TMEM2; gene symbol *CEMIP2*) is such a hyaluronidase (25, 26). Our results revealed that TMEM2 is a type II transmembrane protein, with the hyaluronidase activity residing in its large extracellular domain (ECD). TMEM2 degrades high–molecular weight HA into fragments as small as ∼5 kDa with a near-neutral pH optimum (25). These properties are consistent with those expected of a hyaluronidase that physiologically functions on the cell surface, prompting us to investigate the cell biological function of TMEM2 in cellular activities such as cell adhesion and migration.

In this study, we show that a variety of tumor cells exhibit the ability to eliminate substrate-immobilized HA in a pattern similar to the distribution of focal adhesions (FAs) and stress fibers, and that this FA-targeted HA degradation is mediated predominantly by TMEM2. TMEM2 depletion inhibits adhesion and migration of tumor cells in an HA-rich environment, phenomena that are accompanied by the reduction in the number and size of FA. Consistent with the localization of hyaluronidase activity to FAs, the TMEM2 protein itself is sequestered at FA sites in tumor cells adhering to HA-containing substrates. Furthermore, we found that TMEM2 directly interacts with α5β1 and other integrins. Our findings demonstrate a critical role for TMEM2-mediated HA degradation in the adhesion and migration of cells on HA-rich ECM substrates and in addition provide novel insight into the early phase of FA formation.

## Results

### TMEM2 degrades substrate-bound HA at FAs

**Figure 1:**
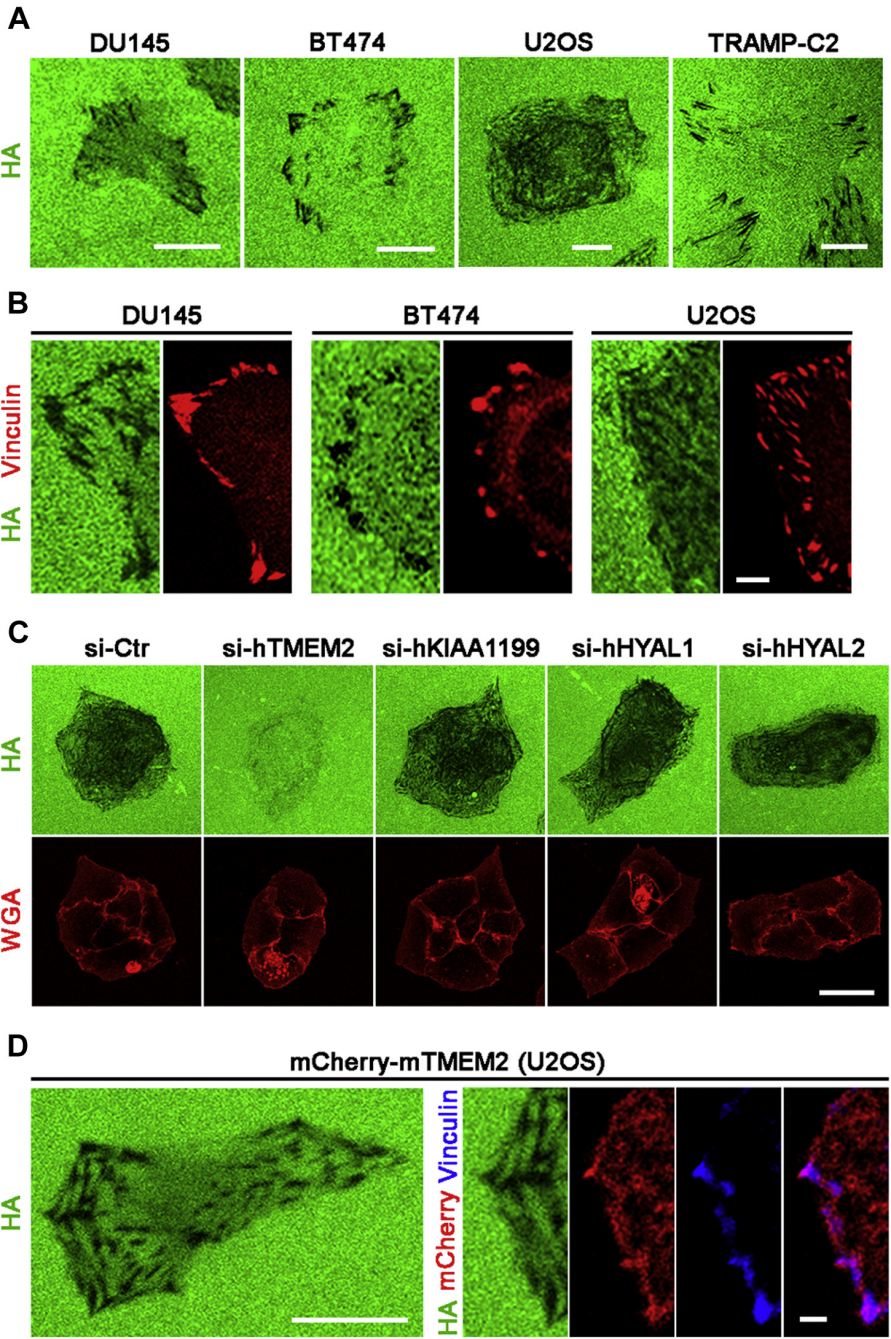
**Tumor cells degrade substrate-bound hyaluronan (HA) at focal adhesions (FAs).***A*, contact-dependent *in situ* degradation of substrate-immobilized HA by DU145, BT474, U2OS, and TRAMP-C2 cells. Cells were cultured for 16 h on the FA-HA substrate as described in Experimental procedures section. *In situ* HA degradation activity is detected as *black* areas in the fluorescent substratum. The scale bar represents 5 μm. *B*, colocalization of vinculin and sites of HA degradation. Cells cultured on the FA-HA substrate were immunostained with anti-vinculin antibody. The scale bar represents 2 μm. *C*, transmembrane protein 2 (TMEM2) is the predominant hyaluronidase that mediates contact-dependent *in situ* HA degradation. Hyaluronidases expressed in U2OS cells (TMEM2, KIAA1199, HYAL1, and HYAL2; see Table 1 for expression levels) were individually knocked down by siRNA treatment, and *in situ* HA degradation assays were performed with siRNA-treated cells. Alexa Fluor 594–conjugated wheat germ agglutinin (WGA) was used to visualize cell morphology. Note that depletion of TMEM2 markedly attenuates *in situ* HA degradation, whereas depletion of other hyaluronidases does not. The scale bar represents 10 μm. *D*, mCherry-fused mouse TMEM2 also induces FA-associated *in situ* HA degradation. U2OS cells stably expressing mCherry-mTMEM2 (endogenous human TMEM is depleted by siRNA treatment) were examined *via in situ* HA degradation assays and immunostained for vinculin. Note that mCherry-mTMEM2 creates a pattern of *in situ* HA degradation indistinguishable from that created by endogenous TMEM2 (*left panel*), and that mCherry signals are present at the sites of HA removal and vinculin immunoreactivities (*right panels*). The scale bar represents 10 μm (*left panel*) and 2 μm (*right panels*).

To analyze cell surface–associated HA degrading activities, we have previously devised an assay in which cells are cultured on a glass substrate immobilized with fluorescein-labeled HA (FA-HA) (25). Using this assay system (referred to as *in situ* HA degradation assay), we examined endogenous HA degrading activity in a variety of tumor cell lines (U2OS human osteosarcoma, BT474 human breast ductal carcinoma, DU145 human prostate adenocarcinoma, and TRAMP-C2 mouse adenocarcinoma) that express high levels of TMEM2 relative to untransformed skin and lung fibroblasts (Table 1). Interestingly, these tumor cells eliminate substrate-immobilized HA in a pattern that resembles the distribution of FAs and stress fibers within the cells (Fig. 1*A*). Immunostaining of these cells for vinculin, a marker for FAs, reveals that the black spots representing sites of HA degradation coincide with vinculin immunoreactivities (Fig. 1*B*). The streak-like pattern of HA degradation likely reflects the movement of FAs during the course of cell adhesion and migration, recording the “history,” rather than instantaneous state, of the degradation of matrix-associated HA.

**Table 1:** Transcript copy numbers of hyaluronidases in various cell lines

Cell line	TMEM2	KIAA1199	HYAL1	HYAL2
U2OS	1.06 × 10^8^ ± 1.16 × 10^6^	9.46 × 10^5^ ± 4.24 × 10^4^	8.58 × 10^4^ ± 0.88 × 10^4^	3.05 × 10^7^ ± 0.85 × 10^6^
BT474	0.61 × 10^8^ ± 3.37 × 10^6^	2.06 × 10^5^ ± 2.58 × 10^4^	4.92 × 10^4^ ± 1.00 × 10^4^	3.24 × 10^7^ ± 1.02 × 10^6^
DU145	1.19 × 10^8^ ± 9.40 × 10^6^	1.78 × 10^5^ ± 1.18 × 10^4^	2.59 × 10^4^ ± 1.52 × 10^4^	2.52 × 10^7^ ± 1.09 × 10^6^
TRAMP-C2	0.51 × 10^8^ ± 1.42 × 10^7^	2.44 × 10^6^ ± 1.14 × 10^5^	4.00 × 10^4^ ± 0.86 × 10^4^	0.87 × 10^7^ ± 3.27 × 10^5^
Skin fibroblast	3.22 × 10^5^ ± 4.09 × 10^4^	2.31 × 10^7^ ± 6.91 × 10^5^	ND	1.79 × 10^6^ ± 0.90 × 10^5^
Lung fibroblast	2.48 × 10^5^ ± 3.52 × 10^4^	5.62 × 10^7^ ± 2.47 × 10^6^	ND	1.61 × 10^6^ ± 0.89 × 10^5^

ND, not detected.

Transcript copy numbers were determined by TaqMan gene expression assay with standard curves generated by reference plasmids. Data represent transcript copy numbers per microgram total RNA shown as mean ± SD from three biological replicates.

Since other hyaluronidases are either intracellular or secretory proteins, and since TMEM2 is the only known cell surface hyaluronidase, TMEM2 seems likely to be responsible for the *in situ* HA degrading activity of these tumor cells. To confirm that this is indeed the case, we used siRNA-mediated knockdown to deplete TMEM2 and other hyaluronidase proteins in U2OS cells and examined the effects of TMEM2 loss on *in situ* HA degradation. siRNAs used in this study decreased respective mRNA levels by 90% to 95% (Fig. S1). Knockdown of TMEM2 almost entirely inhibited *in situ* HA degradation (Fig. 1*C*). On the other hand, knockdown of other hyaluronidases (HYAL1, HYAL2, and KIAA1199) had little effect on *in situ* HA degradation (Fig. 1*C*), indicating that TMEM2 is the hyaluronidase primarily responsible for contact-dependent HA degradation.

These results further suggest that TMEM2 hyaluronidase activity is present in association with FAs at sites of *in situ* HA degradation. To directly confirm the presence of TMEM2 protein at these sites, we established U2OS cells stably expressing mCherry-tagged full-length mouse TMEM2 (referred to as mCherry-mTMEM2 cells). Following depletion of endogenous human TMEM2 in these cells using siRNA specific for human TMEM2, we examined their pattern of *in situ* HA degradation. mCherry-mTMEM2 cells exhibit a pattern of *in situ* HA degradation similar to that seen with parental U2OS cells (Fig. 1*D*, *left panel*). Immunostaining of these cells for vinculin demonstrates that mCherry signals exhibit overlapping colocalization with both vinculin-positive puncta and sites of HA removal (Fig. 1*D*, *right panels*). Together, these results demonstrate that TMEM2 is the predominant, if not the sole, hyaluronidase responsible for FA-associated HA degradation and suggest a potential role for TMEM2 in FA assembly or function in the context of cell adhesion on complex HA-containing substrates.

### TMEM2 is required for efficient cell adhesion and migration on HA-rich substrates

**Figure 2:**
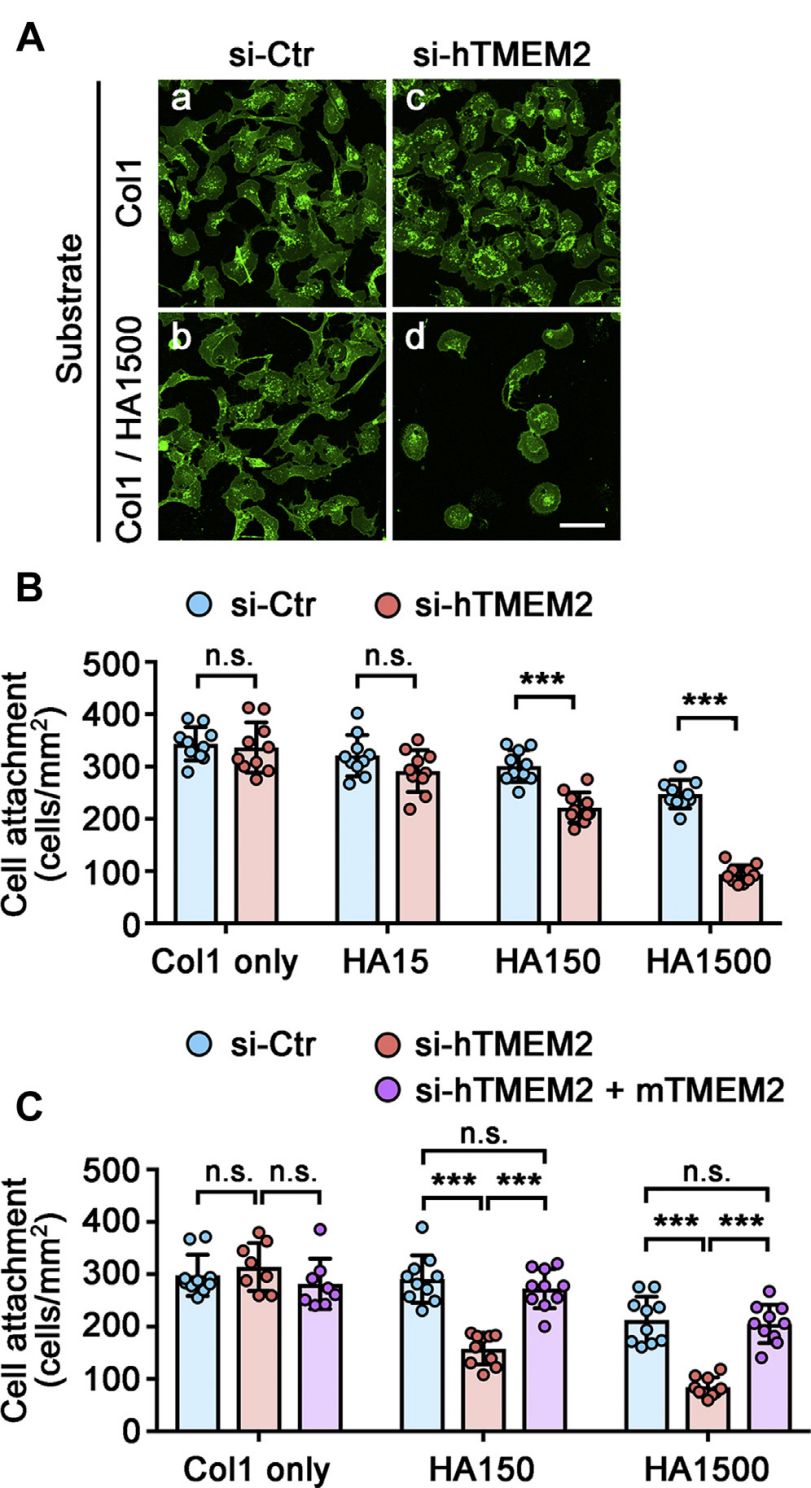
**Transmembrane protein 2 (TMEM2) expression is necessary for efficient adhesion of U2OS cells to hyaluronan (HA)-containing substrates.***A*, representative images of U2OS cell adhesion to the Col1 (*a* and *c*) and Col1/HA1500 mixed substrate (*b* and *d*). Control (*a* and *b*) and TMEM2-depleted (*c* and *d*) cells were allowed to adhere to the substrate for 6 h and stained with Alexa 488-wheat germ agglutinin. The scale bar represents 10 μm. *B*, quantitative analysis of U2OS cell adhesion to the Col1/HA mixed substrates following siRNA-mediated TMEM2 depletion. Coverslips were coated with Col1 (*Col1 only*), Col1 and HA15 (*HA15*), Col1 and HA150 (*HA150*), or Col1 and HA1500 (*HA1500*), and adhesion assays were performed as described in Experimental procedures section. Data are shown as mean ± SD (n = 10 per condition). ∗∗∗*p* < 0.001; n.s., not significant by two-way ANOVA with Bonferroni's multiple comparison test. *Brackets* indicate comparisons between control (*blue*) and TMEM2 siRNA-treated (*red*) cells. Note that siRNA-mediated TMEM2 depletion inhibits cell adhesion to mixed Col1/HA substrates, and that the inhibitory effect of HA is dependent on HA size. *C*, confirmation of the specificity of siRNA effects. We examined the ability of mouse TMEM2, which does not contain the human TMEM2 target sequence, to rescue the effect of human TMEM2 knockdown on cell adhesion. Adhesion assays were performed as described previously for parental U2OS cells treated with control siRNA (*blue*), parental U2OS cells treated with siRNA against human TMEM2 (*red*), and mouse TMEM2-transfected U2OS cells (mCherry-mTMEM2 cells) treated with siRNA against human TMEM2 (*purple*). Data are shown as mean ± SD (n = 8–10 per condition). ∗∗∗*p* < 0.001; n.s., not significant by two-way ANOVA with Bonferroni's multiple comparison test.

While the mechanistic process of integrin-mediated cell–substrate adhesion has been resolved in great detail, the role of nonadhesive or antiadhesive ECM components in cell adhesion remains incompletely addressed. Since excess HA is inhibitory to cell adhesion (10), it has been suggested that HA needs to be remodeled or removed prior to the establishment of firm engagement between integrins and their matrix ligands (27, 28, 29). However, potential molecular mechanisms by which HA is remodeled by cells have been poorly investigated. We hypothesize that TMEM2 is the key endogenous hyaluronidase that remodels and/or removes HA during the process of cell adhesion to complex ECM substrata. To test this hypothesis, we examined the adhesion of U2OS cells to mixed substrates consisting of type I collagen (Col1) and HA of various molecular sizes (HA1500 [1,500,000–1,750,000 Da], HA150 [130,000–150,000 Da], and HA15 [8000–15,000 Da]; see Experimental procedures section for detail) with or without siRNA-mediated TMEM2 knockdown. Control U2OS cells (treated with negative control siRNA) adhere equally well to a homogeneous Col1 substrate and a mixed Col1/HA1500 substrate (Fig. 2*A*, a and b). However, adhesion of TMEM2-depleted U2OS cells to the Col1/HA1500 substrate is greatly impaired, even though adhesion of these cells to the homogeneous Col1 substrate is not affected (Fig. 2*A*, c and d), indicating that TMEM2 expression is needed for efficient adhesion to HA-containing substrates. We then examined the effect of the molecular size of HA on U2OS cell adhesion. On the mixed Col1/HA15 substrate, TMEM2 depleted and control cells attach equally well, indicating that low–molecular weight HA has little antiadhesive effect and, accordingly, that the expression of TMEM2 is not needed to promote adhesion (Fig. 2*B*). In contrast, TMEM2 expression makes a significant difference for cell adhesion to mixed substrates containing HA of higher molecular sizes. On the Col1/HA1500 substrate, TMEM2 depletion results in more than a 60% reduction in the number of attached cells in comparison with controls (Fig. 2*B*). On the Col1/HA150 substrate, the effect of TMEM2 depletion is smaller than that on the Col1/HA1500 kDa substrate, but the number of attached cells is still significantly reduced by TMEM2 depletion (Fig. 2*B*). These effects of TMEM2 depletion on cell adhesion are almost completely restored in the knockdown cells by the expression of mouse TMEM2, which does not contain the siRNA target sequence (Fig. 2*C*). This confirms the specificity of the siRNA effects.

**Figure 3:**
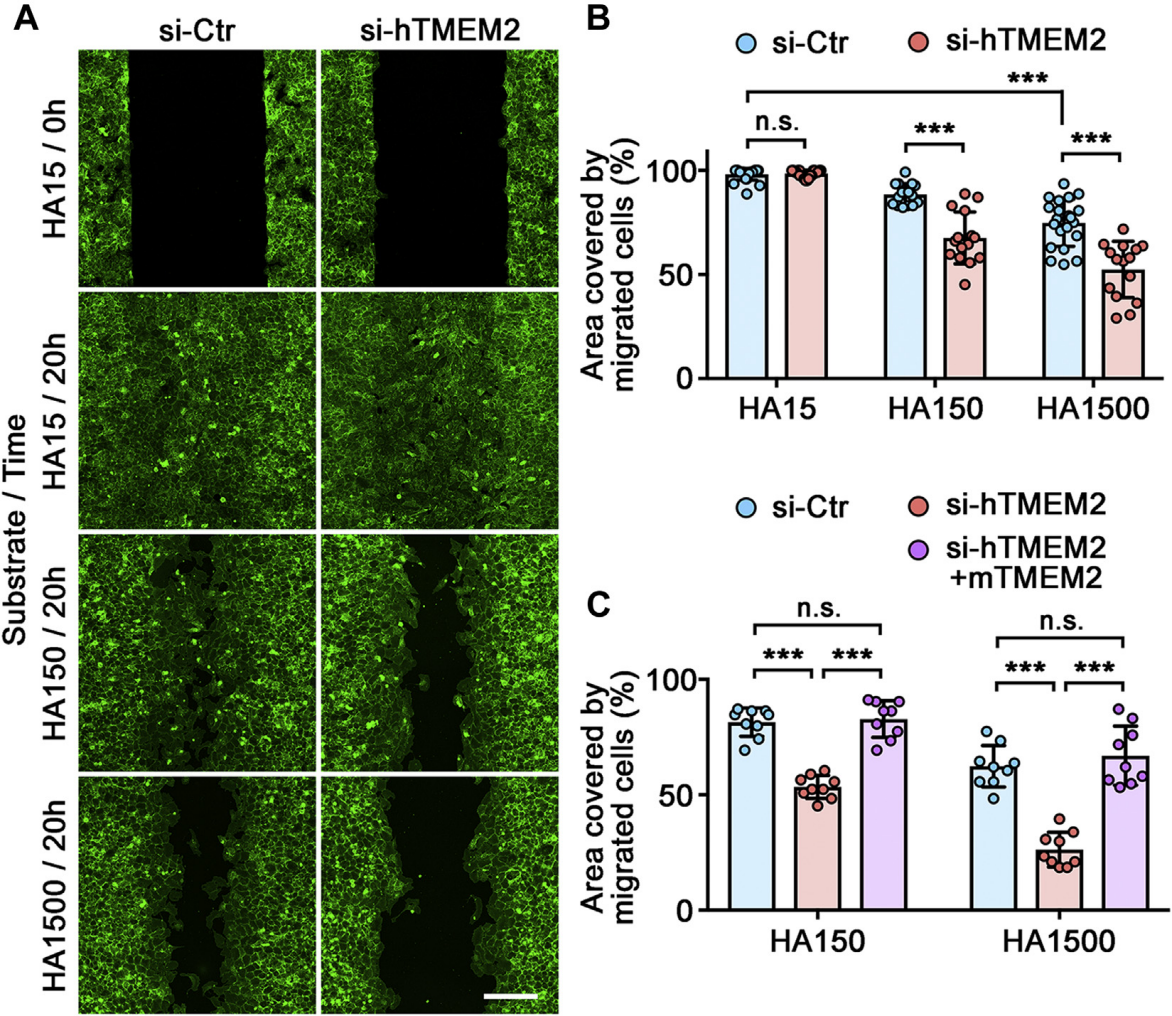
**Transmembrane protein 2 (TMEM2) is required for efficient cell migration on hyaluronan (HA)-containing substrates.***A*, representative images of the migration of TMEM2-depleted and control U2OS cells into a cell-free gap on mixed substrates of Col1 and HA. *Top panels* (*HA15/0 h*) show images of gaps immediately after removal of the ibidi 2-well Culture-Insert. Other panels show images of gaps after a 20 h incubation on Col1/HA15, Col1/HA150, and Col1/HA1500 substrates. The scale bar represents 200 μm. *B*, quantitative analysis of cell migration. Data represent the mean ± SD of the gap area covered by migratory cells relative to the area of the original gap (n = 15–24 per condition). ∗∗∗*p* < 0.001; n.s., not significant by two-way ANOVA with Bonferroni's multiple comparison test. *C*, confirmation of the specificity of siRNA effects. Migration assays were performed for parental U2OS cells treated with control siRNA (*blue*), parental U2OS cells treated with siRNA against human TMEM2 (*red*), and mouse TMEM2-transfected U2OS cells (mCherry-mTMEM2 cells) treated with siRNA against human TMEM2 (*purple*). Data represent mean ± SD (n = 9 per condition). ∗∗∗*p* < 0.001; n.s., not significant by two-way ANOVA with Bonferroni's multiple comparison test.

Next, we examined the effect of TMEM2 depletion on cell migration in a wound healing–type assay. In order to create a cell-free gap that retains an intact Col1/HA coating, we used 2-well silicone wound healing chambers (Culture-Insert 2-Well; ibidi). Unlike the gap creation by scratching, this method of gap creation does not damage the Col1/HA coating in the gap (see Experimental procedures section for the detailed method). Cell migration into gaps of the Col1/HA15 substrate is not significantly different between TMEM2-depleted and control cells (*HA15/20 h* in Fig. 3*A*; also *HA15* in Fig. 3*B*). On the other hand, migration into gaps on the Col1/HA150 and Col1/HA1500 substrates is significantly attenuated in TMEM2-depleted cells compared with control-treated cells (*HA150/20 h* and *HA1500/20 h* in Fig. 3*A*; also *HA150* and *HA1500* in Fig. 3*B*). As in the case of cell adhesion, expression of mouse TMEM2 in human TMEM2-depleted cells fully restores their ability to migrate into gaps on the high–molecular weight HA substrates (*si-hTMEM2 + mTMEM2* in Fig. 3*C*), confirming both the involvement of TMEM2 and the specificity of the siRNA effects. Together, these results demonstrate that TMEM2 plays a critical role in promoting cell adhesion and migration on HA substrates.

### Role of TMEM2 in the development of FAs

**Figure 4:**
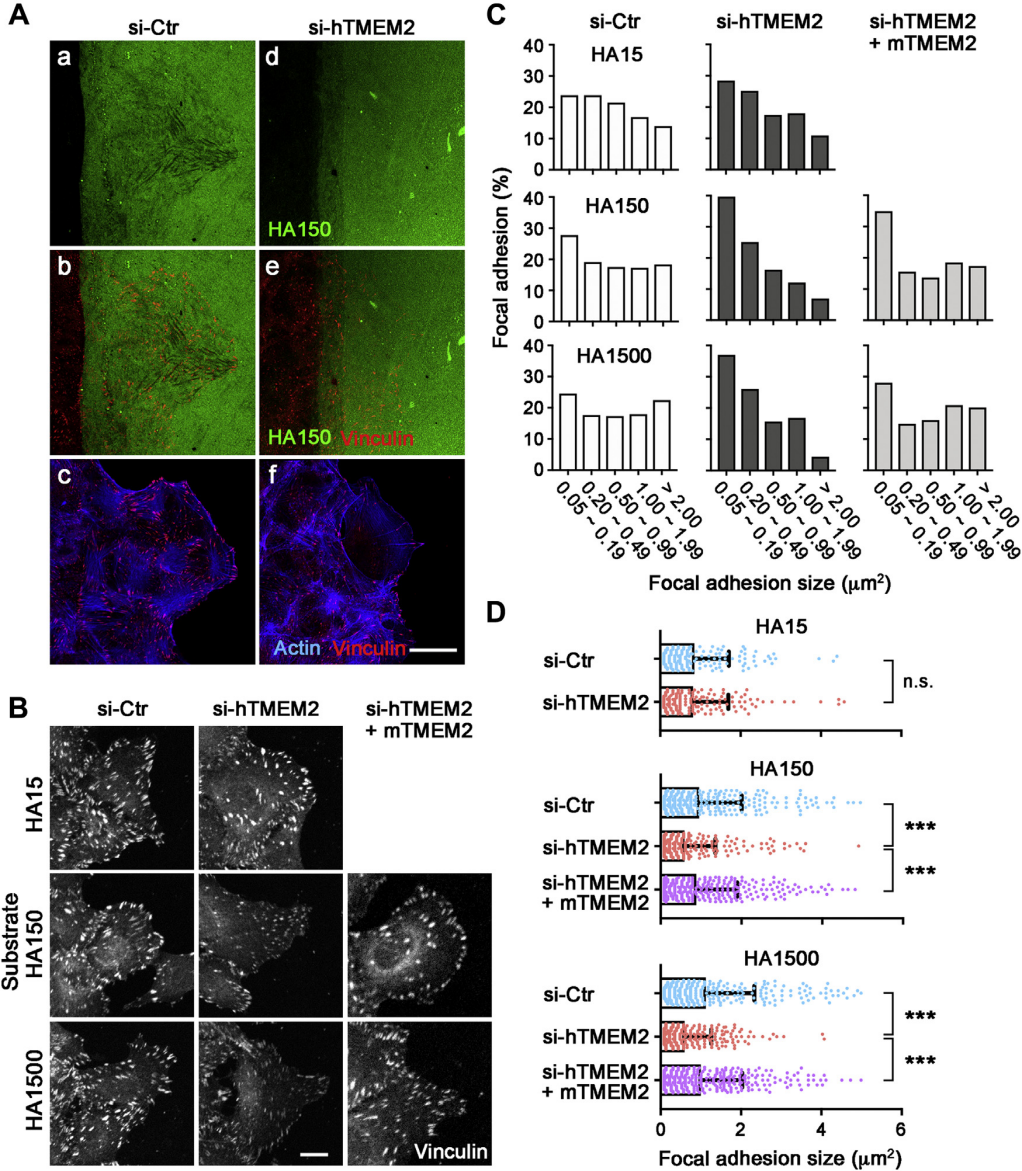
**Transmembrane protein 2 (TMEM2) is required for the efficient formation of focal adhesions (FAs) in cells migrating on hyaluronan (HA)-containing substrates.***A*, representative images of *in situ* HA degradation and FA formation by U2OS cells migrating on Col1/FA-HA mixed substrates (*green*). U2OS cells were either treated with control siRNA (a, b, and c) or siRNA to TMEM2 (d, e, and f). Cells were double stained for vinculin (*red*) and F-actin (*blue*). (a and d) *in situ* HA degradation patterns; (b and e) distribution of vinculin-immunoreactive FAs in relation to sites of HA degradation; and (c and f) distribution of FAs and F-actin. The scale bar represents 10 μm. *B*, FA formation in cells at the forefront of migration. U2OS cells migrating on Col1/HA mixed substrates were immunostained for vinculin. Migration assays were performed with parental U2OS cells treated with control siRNA (*si-Ctr*; *left panels*), parental U2OS cells treated with siRNA against human TMEM2 (*si-hTMEM2*; *middle panels*), and mouse TMEM2-transfected U2OS cells treated with siRNA against human TMEM2 (*si-hTMEM2 + mTMEM2*; *right panels*). The scale bar represents 5 μm. *C*, FA size distribution in cells at the forefront of migration as shown in *B*. Histogram was derived from the measurements of >300 FAs per condition pooled from triplicate experiments. *D*, average size of FAs in cells at the forefront of migration. Data represent mean ± SD (number of FAs analyzed: >300 per condition pooled from three independent experiments). ∗∗∗*p* < 0.001; n.s., not significant by one-way ANOVA with Bonferroni's multiple comparison test.

FAs play a pivotal role in cell migration on two-dimensional substrates (30). We therefore used the wound healing assay to examine the respective patterns of *in situ* HA degradation and FA formation by migrating U2OS cells. In control U2OS cultures, migrating cells exhibit robust *in situ* HA degradation, with cells at the migratory forefront creating sharp streaks of HA-deficient substratum (Fig. 4*A*, a). Immunostaining for vinculin in these cultures reveals cells undergoing robust formation of FAs along these HA degradation streaks (Fig. 4*A*, b). In TMEM2-depleted U2OS cells, in contrast, few sites of HA degradation are observed (Fig. 4*A*, d), and the number of vinculin-immunoreactive FAs is greatly diminished compared with control U2OS cells (Fig. 4*A*, e). Double staining for F-actin and vinculin reveals greatly reduced formation of FA and stress fibers in TMEM2-depleted cells at the migratory forefront (Fig. 4*A*, c and f). We also analyzed FAs in TMEM2-depleted and control cells migrating on substrates of different HA sizes. On the Col1/HA150 and Col1/HA1500 substrates, TMEM2-depleted cells exhibit impaired FA formation, with significant reductions in both FA size and the intensity of vinculin immunoreactivity (*HA150* and *HA1500* in Fig. 4*B*). On the HA15 substrate, there are no apparent differences in the distribution and size of FAs between TMEM2-depleted and control cells (*HA15* in Fig. 4*B*). Quantification of the area of FAs confirms these observations. Control U2OS cells on the Col1/HA150 and Col1/HA1500 substrates exhibit an even distribution of FA sizes, ranging from >2 μm^2^ to 0.05 μm^2^ (*si-Ctr* in Fig. 4*C*). In contrast, the majority of FAs observed in TMEM2-depleted cells have sizes less than 0.5 μm^2^ (*si-hTMEM2* in Fig. 4*C*). The average FA size in TMEM2-depleted cells is significantly smaller than that seen in control cells on both Col1/HA150 and Col1/HA1500 substrates (Fig. 4*D*). The involvement of TMEM2 and the specificity of the siRNA effects against endogenous human TMEM2 are confirmed by the restoration of FA formation by expression of mouse TMEM2 (*si-hTMEM2 + mTMEM2* in Fig. 4, *B*–*D*). Taken together, these results indicate that TMEM2 plays a functional role in promoting FA development on HA-containing substrates.

### TMEM2 binds directly to integrins *via* interactions between the ECDs

**Figure 5:**
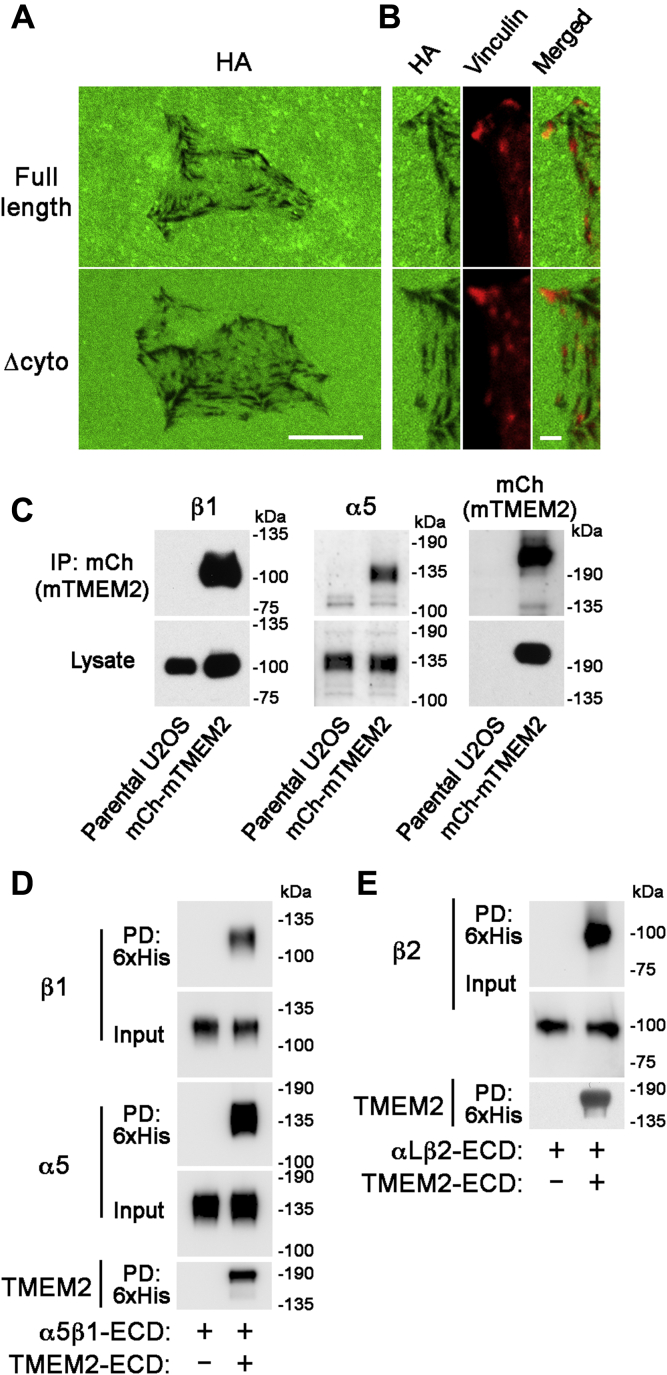
**Association of transmembrane protein 2 (TMEM2) with integrins *via* interactions between the extracellular domains.***A* and *B*, targeting of TMEM2 to focal adhesions (FAs) does not require the cytoplasmic domain of TMEM2. In this experiment, mCherry-mTMEM2 (*full length*) and mCherry-mTMEM2/Δcyto (Δ*cyto*) cells were analyzed for their *in situ* hyaluronan (HA) degradation activities. To allow specific analysis of the activity of the full-length mouse TMEM2 and its Δcyto deletion mutant, expression of endogenous human TMEM2 was silenced by siRNA treatment prior to the assay. *A*, *in situ* HA degradation assays were performed on substrate immobilized with FA-HA, as described in Experimental procedures section. Note that the pattern of *in situ* HA degradation is indistinguishable between mCherry-mTMEM2/Δcyto and mCherry-mTMEM2 cells. The scale bar represents 10 μm. *B*, immunostaining for vinculin in mCherry-mTMEM2 and mCherry-mTMEM2/Δcyto cells on the FA-HA substrate. Note that the sites of HA degradation colocalize with vinculin-immunoreactive puncta in both mCherry-mTMEM2/Δcyto and mCherry-mTMEM2 cells. The scale bar represents 2 μm. *C*–*E*, TMEM2 associates with integrins *via* extracellular interactions. *C*, cell surface–expressed TMEM2 is coimmunoprecipitated with integrin α5β1. mCherry-mTMEM2 cells were treated with the membrane-impermeable crosslinker 3',3'-dithiobis(sulfosuccinimidyl propionate), and the lysates from these cells were immunoprecipitated with anti-mCherry antibody, followed by immunoblotting analysis with antibodies to α5, β1, and mCherry. *D*, the extracellular domain of TMEM2 directly binds α5β1. Binding between TMEM2 extracellular domain (ECD) and the α5β1 ECD heterodimer was analyzed by a pull-down assay. *E*, the ECD of TMEM2 directly binds αLβ2 (lymphocyte function-associated antigen-1). Binding between TMEM2 ECD and the αLβ2 ECD heterodimer was analyzed by a pull-down assay.

Colocalization of TMEM2-mediated HA degradation at FA sites (Fig. 1) suggests that TMEM2 may physically associate with some FA component(s). Since TMEM2 is a transmembrane protein, we first explored the possibility that TMEM2 might interact with FA-associated cytoplasmic/cytoskeletal proteins *via* its cytoplasmic domain. However, coimmunoprecipitation assays using human embryonic kidney 293 (HEK293) cells failed to reveal specific interactions of TMEM2 with major FA-associated cytoplasmic proteins, including talin and focal adhesion kinase (not shown). To examine more directly whether the cytoplasmic domain is required for targeting TMEM2-mediated *in situ* HA degradation to FAs, we prepared an expression construct for mCherry-tagged mouse TMEM2 that lacks the entire cytoplasmic domain (but retains the transmembrane domain) and established stably transfected U2OS cells (referred to as mCherry-mTMEM2/Δcyto cells). As shown in Figure 5*A*, mCherry-mTMEM2/Δcyto cells exhibit a pattern of *in situ* HA degradation that is indistinguishable from that seen with control mCherry-mTMEM2 cells expressing full-length mouse TMEM2. Furthermore, in both mCherry-mTMEM2/Δcyto and mCherry-mTMEM2 cells, vinculin-immunoreactive puncta are localized at the sites of *in situ* HA degradation (Fig. 5*B*). Taken together, these results demonstrate that the cytoplasmic domain of TMEM2 is not required for TMEM2-dependent and FA-associated *in situ* HA degradation. Instead, the recruitment of TMEM2 proteins to FA sites seems likely to be mediated by interactions between their ECDs.

Integrins have been shown to interact with a variety of membrane-associated proteins *via* interactions between ECDs (31, 32, 33, 34, 35). We therefore used two independent assays to examine whether TMEM2 interacts with α5β1 integrin, a dominant integrin expressed in U2OS cells. First, association of TMEM2 and α5β1 on the cell surface was examined by coimmunoprecipitation following the crosslinking of cell surface proteins. mCherry-mTMEM2 cells were treated with the membrane-impermeable crosslinker 3',3'-dithiobis(sulfosuccinimidyl propionate) (DTSSP), and lysates from DTSSP-treated cells were immunoprecipitated with anti-mCherry antibody. Association of α5β1 with TMEM2 was analyzed by immunoblotting with anti-α5 and anti-β1 antibodies. As shown in Figure 5*C*, α5β1 is coimmunoprecipitated with TMEM2, indicating that TMEM2 and α5β1 are in direct contact or in close proximity on the cell surface. Second, to examine whether TMEM2 and α5β1 directly interact *via* their ECDs, we produced 6x His-tagged TMEM2 ECD in HEK293 cells and tested its binding to recombinant human α5β1 ECD heterodimer in a pull-down assay. As shown in Figure 5*D*, α5β1 ECD is specifically pulled down with ProBond resin loaded with TMEM2 ECD, demonstrating direct interaction between the ECDs of TMEM2 and α5β1. Finally, to explore whether TMEM2 can also interact with other integrins, we examined TMEM2 ECD binding to the major lymphocyte integrin αLβ2 (lymphocyte function-associated antigen-1). As in the case of α5β1, the pull-down assay demonstrates direct binding between TMEM2 ECD and the αLβ2 ECD heterodimer (Fig. 5*E*). Together, these results demonstrate that TMEM2 binds directly to integrins *via* interactions between the respective ECDs, consistent with the concept that this interaction forms the basis for TMEM2 localization to FA sites.

## Discussion

Although studies on cell–matrix adhesion have focused mainly on integrins and their protein ligands, ECM *in vivo* also contains large amounts of various glycosaminoglycans. Among these, HA is of particular importance because of its abundance, peculiar biophysical properties, and biological effects on cell adhesion and migration. The mechanisms by which cells control the effects of this voluminous polysaccharide, as a means of modulating cell adhesion and migration, remain poorly understood. In this article, we demonstrate that adherent cells degrade matrix-associated HA *via* the action of the cell surface hyaluronidase TMEM2, and that this activity is necessary for cells to achieve robust cell adhesion and migration on HA-containing substrates. Significantly, our study demonstrates that TMEM2-mediated HA degradation colocalizes with FAs, and that TMEM2 directly associates with integrins, indicating that *in situ* HA degradation and FA formation are coordinated during the process of cell adhesion.

While increased tumor cell degradation of HA has previously been implicated in tumor progression (36, 37), little attention has been paid to the mechanisms by which tumor cells degrade HA in the microenvironment surrounding them. Our assay system using substrate-immobilized HA allows us to interrogate cell surface–associated HA degrading activities (25). Intriguingly, our results revealed that many tumor cells degrade substrate-bound HA in association with FA sites. siRNA-mediated knockdown experiments show that this contact-dependent *in situ* HA degradation is predominantly mediated by TMEM2, rather than by other intracellular and secretory hyaluronidases. This observation appears to be consistent with the fact that TMEM2 is the only known transmembrane hyaluronidase. Further supporting the role of TMEM2 in FA-associated HA degradation, we have shown that TMEM2 proteins are colocalized with FAs, and moreover, physically interact with integrins. Consistent with our findings, it is noteworthy that a previous proteomic profiling of FA-associated proteins reported by Kuo *et al.* (38) has identified TMEM2 as one of the proteins detected in FAs from fibroblasts (human foreskin fibroblast-1). Taken together, these observations demonstrate that cells degrade matrix-associated HA at FA sites, and that this localized HA degradation is mediated predominantly by TMEM2. ECM degradation coinciding with FAs has also been demonstrated for the membrane-type 1 matrix metalloproteinase (39). Thus, like membrane-type 1 matrix metalloproteinase, TMEM2 can be regarded as an ECM-degrading enzyme used by cells to remodel the ECM to promote adhesion and migration.

Another key finding of this study is that TMEM2 binds to α5β1 integrin *via* an interaction between the respective ECDs of the two proteins (Fig. 5, *C* and *D*). We also find that this interaction is not limited to α5β1; at least one other integrin, αLβ2, also interacts with TMEM2 *via* its ECDs (Fig. 5*E*). The interaction with α5β1 likely plays the primary role in the localization of TMEM2 to FAs in U2OS cells. Furthermore, the fact that the TMEM2/Δcyto mutant can drive FA-associated *in situ* HA degradation in the same manner as full-length TMEM2 (Fig. 5*A*) suggests that the extracellular TMEM2–integrin interaction is sufficient to drive FA-associated *in situ* HA degradation by TMEM2. There are multiple functionally important proteins that associate with integrins *via* extracellular interactions. Examples include tetraspanins, integrin-associated protein/CD47, a disintegrin and metalloproteinase 17, and CD16B (31, 32, 33, 34, 35). At present, we have not determined the binding sites involved in the TMEM2–integrin interaction because of difficulties in expressing some deletion mutants of TMEM2.

**Figure 6:**
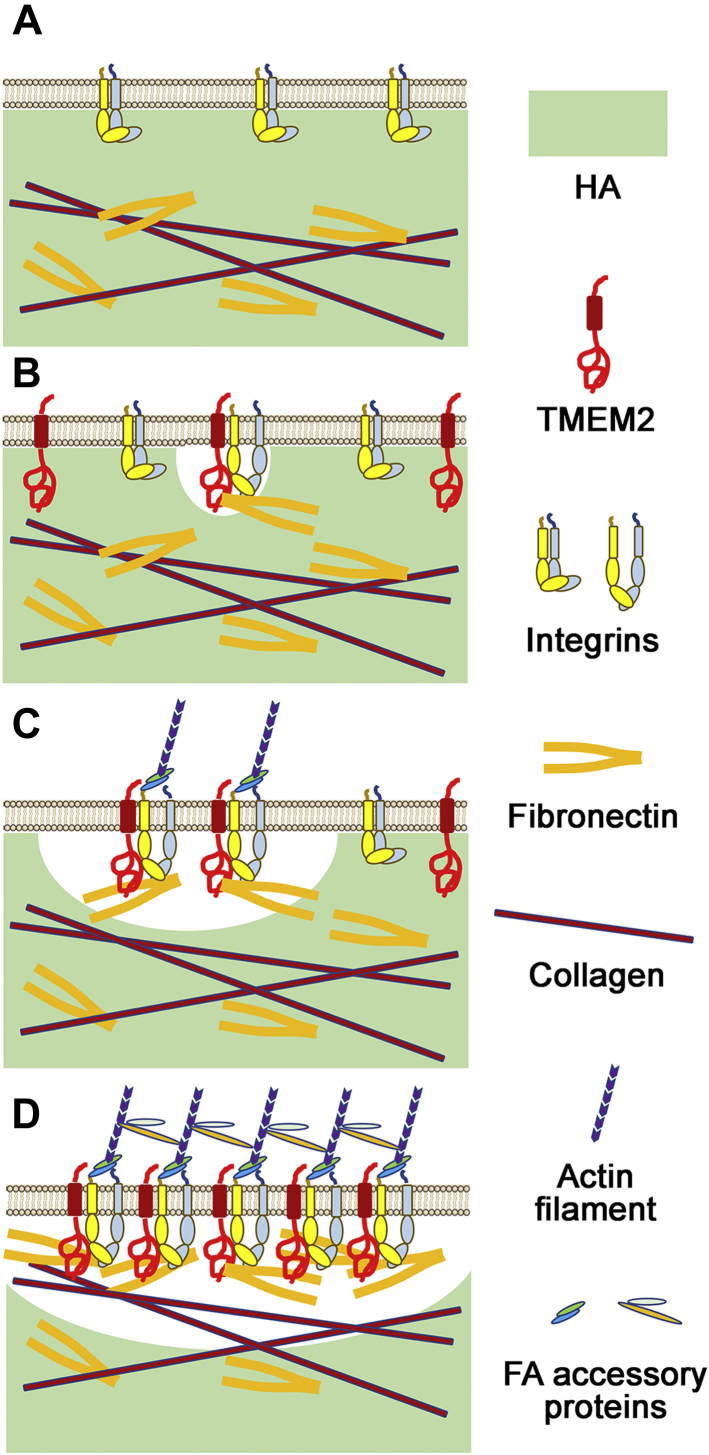
**A model for the role of transmembrane protein 2 (TMEM2) in integrin-mediated cell adhesion and migration.** Our results suggest that TMEM2-dependent degradation of hyaluronan (HA) is critical for cells to form strong cell–matrix adhesion on HA-rich extracellular matrix (ECM). *A*, high levels of HA in the ECM are inhibitory to the direct engagement of integrins to their ECM ligands. *B*, in the presence of TMEM2, HA in the ECM is locally removed, which generates a microenvironment that is permissible to the direct integrin–ECM engagement. *C*, the association between TMEM2 and integrins promotes the FA formation and maturation *via* further removal of HA in the vicinity of the integrin–ECM engagement. *D*, this in turn facilitates integrin clustering, integrin-mediated downstream signaling, and cellular responses. See the text for further discussion.

The effect of TMEM2 knockdown demonstrates the functional importance of TMEM2 in cell adhesion and migration. Clearly, the expression of TMEM2 is necessary for cells to form robust adhesion to substrate containing high–molecular weight HA. The precise mechanistic process by which TMEM2 is functionally involved in FA formation is of great interest in understanding how cells achieve robust adhesion and migration on HA-containing substrates. Figure 6 depicts a model for the role of TMEM2 in integrin-mediated cell adhesion, based on the observations made in this study. The central tenet of the model is that TMEM2 removes HA in the vicinity of integrin-mediated cell–matrix adhesion, thereby promoting integrin–ligand engagement and FA formation and maturation. An important question remaining to be addressed is whether TMEM2-mediated HA degradation *precedes* or *follows* FA formation. In other words, are FAs formed at sites where TMEM2 has removed HA or is TMEM2 recruited to preformed FAs? The observation that TMEM2 knockdown attenuates FA formation suggests that TMEM2 function may be a prerequisite for FA formation. Nevertheless, it is possible that the actual process represents a compromise between these two extreme possibilities—TMEM2 may be recruited to sites of early FAs (*e.g.*, nascent adhesions or focal complexes), and then TMEM2-mediated HA removal at these nascent adhesion sites may facilitate further ECM–integrin interaction and the development of these initial adhesions into mature FAs. In this context, it is noteworthy that Zaidel-Bar *et al.* (28) observed in chondrocytes that initial HA-mediated soft cell–matrix contacts are replaced within 1 min by early integrin-containing FAs. It is conceivable that TMEM2 is the key functional entity in this process that enables the progression of initial soft contacts to early and eventually mature FAs. In any case, the precise mechanistic process of early cell adhesion and the role of TMEM2 in the process will need to be addressed *via* use of high-resolution live cell imaging.

The cell biological function of TMEM2 demonstrated in this article has potential clinical relevance to cancer. HA is highly accumulated in tumor stroma, especially in high-grade cancers characterized by desmoplastic changes (11, 12, 16, 40, 41). In tumor stroma, HA is produced predominantly by stromal cells, rather than by neoplastic tumor cells (13, 14, 15, 16), and HA-dense ECM represents a hostile microenvironment for resident tumor cells (12, 42). On the other hand, tissue microarray data, deposited in The Human Protein Atlas (www.proteinatlas.org), demonstrate that TMEM2 is predominantly expressed in the tumor parenchyma of breast, lung, liver, colorectal, and prostate cancers. These observations suggest the possibility that high-grade tumor cells may use TMEM2 to degrade stromal HA, thereby remodeling the surrounding microenvironment in a way that is favorable for their adhesion, migration, and invasion. In this context, it is particularly interesting to note that *TMEM2* is one of the three genes in human breast cancer whose expression is highly upregulated by SOX4, a presumed driver of tumor invasion, and, furthermore, that TMEM2 expression correlates significantly with reduced overall patient survival in high-risk breast cancers (43). High TMEM2 expression is also significantly correlated with reduced overall survival of patients with renal cancer (www.proteinatlas.org). These clinical correlation data are intriguing in terms of their support of a model in which TMEM2 is a key matrix-remodeling enzyme in the context of tumor progression.

## Experimental procedures

### Plasmids and siRNAs

An expression plasmid for mouse full-length TMEM2 fused with mCherry at the N terminus (mCherry-mTMEM2) was generated as follows: An AgeI site was created between the T7 promoter and the KpnI site upstream of mouse TMEM2 complementary DNA (cDNA) in pcDNA3 by using the QuikChange Site-Directed Mutagenesis Kit (Agilent Technologies). mCherry cDNA was then ligated into AgeI/KpnI-excised TMEM2-pcDNA3 (25). An expression plasmid for the mouse TMEM2 cytoplasmic deletion mutant (mCherry-mTMEM2/Δcyto) was created by deletion of residues Met1 to Thr82 between mCherry and transmembrane domain using QuikChange Mutagenesis Kit. An expression plasmid for the ECD (Ser104–Leu1383) of mouse TMEM2 fused with 6× His was generated by removing the C-terminal Myc peptide from mouse TMEM2–ECD in pSecTag2A (25) using QuikChange Mutagenesis Kit. Sequences of all insert cDNA were confirmed by primer extension sequencing. The following siRNAs were purchased from Bioneer: human TMEM2 (#1153123), KIAA1199 (#1079190), HYAL1 (#1072019), HYAL2 (#1072028), and negative control (#SN1001). Inhibition of mRNA transcription by each siRNA was verified by quantitative PCR as described later. The specificity of siRNA effects in siRNA-treated cells was confirmed by expression of mouse TMEM2, which does not contain the siRNA target sequence, throughout this study.

### Cell culture and transfection

The following cell lines were purchased from American Type Culture Collection: U2OS (human osteosarcoma, HTB-96), BT474 (human breast ductal carcinoma, HTB-20), DU145 (human prostate carcinoma, HTB-81), TRAMP-C2 (mouse prostate adenocarcinoma, CRL-2731). Stable cell lines expressing mCherry-fused mouse TMEM2 (mCherry-mTMEM2) and its cytoplasmic deletion mutant (mCherry-mTMEM2/Δcyto) were generated by transfection of the respective expression constructs using Lipofectamine 3000 (Thermo Fisher Scientific), followed by G418 selection and cell sorting. The following culture media were used for U2OS and TRAMP-C2, Dulbecco's modified Eagle's medium (Corning, 13-010-CV) containing 10% fetal bovine serum (FBS) (Corning, 35-010-CV); for BT474, RPMI1640 containing 10% FBS (Thermo Fisher Scientific, 11875093); for DU145, Eagle's minimal essential medium containing 10% FBS (Corning, 10-009-CV). Transfection of siRNA was performed by using Lipofectamine RNAiMAX (Thermo Fisher Scientific) according to manufacturer's instructions.

### Quantitative PCR

Total RNA was isolated from human cells using an RNeasy Mini Kit (Qiagen), and cDNA was prepared by using SuperScript VILO MasterMix (Thermo Fisher Scientific). The TaqMan gene expression assay was carried out by using a LightCycler 96 system (Roche Applied Science) with the following TaqMan primer/carboxyfluorescein-conjugated probe sets obtained from Applied Biosystems: TMEM2 (Hs00910521), KIAA1199 (Hs01552114), HYAL1 (Hs00201046), HYAL2 (Hs01117343), and GAPDH (Hs02758891). Absolute quantification was performed as described in a previous study (25) using reference plasmids for each gene. Reference plasmids were produced by subcloning of PCR products into pGEM-T Easy Plasmid (Promega), and insert cDNAs were confirmed by sequencing. Reduction of hyaluronidase expression in siRNA-treated U2OS cells compared with control cells was calculated using ΔΔCt method following normalization to GAPDH mRNA.

### *In situ* HA degradation assay

This assay was performed as described previously (25). Briefly, trypsinized cells were seeded on coverslips coated with FA-HA (Cosmo Bio; CSR-FAHA-L2) and incubated for 16 h. For siRNA experiments, cells were treated with siRNA for 3 days before use in this assay. Cells were fixed with 4% paraformaldehyde in PBS for 10 min at RT and stained with Alexa Fluor 594-conjugated wheat germ agglutinin (WGA) (Thermo Fisher Scientific; W11262) diluted 1:100 in Hank's balanced salt solution containing calcium and magnesium (HBSS^++^) for 30 min at RT to visualize cell shape. In some experiments, fixed cells were permeabilized with PBS containing 0.2% Triton X-100 for 10 min and blocked with PBS containing 1% bovine serum albumin (BSA) (IgG-free; Sigma; A2058) for 30 min at RT, followed by immunocytochemistry with mouse monoclonal anti-vinculin antibody (1:200 dilution in 1% BSA–PBS) (Sigma; V9264, clone hVIN-1) and Rhodamine Red-X-conjugated anti-mouse IgG secondary antibody (1:1000 dilution in 1% BSA–PBS) (Jackson ImmunoResearch Laboratories, Inc; 715-295-151). Coverslips were mounted with ProLong Gold Antifade Reagent (Thermo Fisher Scientific; P36934). Fluorescent images were captured using Zeiss LSM710 laser-scanning microscope.

### Cell adhesion assay

Coverslips were coated with type I collagen only (Corning; 354249) or a mix of type I collagen and unlabeled HA specimens of various average molecular weight, namely HA15 (molecular weight range, 8000–15,000; Sigma; 40583), HA150 (130,000–150,000; Sigma; 75043), and HA1500 (1,500,000–1,750,000) (Sigma; 63357). In sequence, glass coverslips were treated with 20% HNO_3_ and then with 0.1 M NaOH. After drying, coverslips were incubated with (3-aminopropyl)triethoxysilane (Sigma; 440140) for 5 min at RT, washed with water 3 times, and treated with 0.25% glutaraldehyde in PBS for 30 min at RT. After washing with PBS, coverslips were coated with 50 μg/ml type I collagen in 0.2 M acetic acid for 2 h at RT. Washed coverslips were then incubated with 0.5 mg/ml unlabeled HA overnight at RT and then blocked with 2% BSA–PBS (heat denatured at 65 °C for 10 min). Trypsinized cells were seeded onto the coated coverslips in 24-well plates at a density of 5 × 10^4^ cells per coverslip and then incubated for 6 h at 37 °C in a CO_2_ incubator. Cells were stained with Alexa Fluor 488–conjugated WGA (Thermo Fisher Scientific; W11261) as described previously. Fluorescent images were captured using Zeiss LSM710 laser-scanning microscope. Cell adhesion was quantified by counting attached cells. Experiments were performed in biological triplicates, and statistical significance was analyzed by two-way ANOVA with Bonferroni's multiple comparison test using GraphPad Prism software (GraphPad Software Inc.).

### Cell migration assay

A wound healing-type assay was performed using 2-well culture inserts (ibidi; 80209) to create a defined 500 μm cell-free gap on Col1/HA substrates. Glass coverslips were coated with type I collagen and HA as described previously. In some experiments, FA-HA (Cosmo Bio; CSR-FAHA-H2) was used in place of unlabeled HA. After drying, 2-well culture inserts (ibidi; 80209) were attached to coated coverslips. Insert-attached coverslips were then transferred into 24-well plates, and on the outside, the inserts were filled with PBS. Cells (1 × 10^4^ cells in 70 μl of culture medium per insert) were seeded into the wells and cultured for 1 day, followed by siRNA treatment within the wells. Three days later, culture inserts were detached from coverslips, and coverslips were transferred into new 24-well plates with fresh culture medium. After a 24 h (for migration assay) or 6 h (for the analysis of FA development) of incubation, cells were stained with Alexa488-WGA or anti-vinculin antibody, as described previously. Fluorescent images were captured by Zeiss LSM710 laser-scanning microscope. Covered area in a gap (0.5 mm in distance) between two wells by migrating cells and size/intensity of vinculin-positive FAs were analyzed by ImageJ 1.51s (NIH). Experiments were performed in biological triplicates, and statistical significance was analyzed by two-way ANOVA with Bonferroni's multiple comparison test using GraphPad Prism software.

### Cell surface crosslinking and immunoprecipitation

mCherry-mTMEM2 cells were cultured for 1.5 h in a 10 cm dish coated with 5 μg/ml fibronectin (Corning; 345008). After washing with HBSS^++^, cells were treated with 1 mM DTSSP (Thermo Scientific; 21578) in HBSS for 30 min at RT. Unreacted reagents were quenched by adding Tris (final concentration, 20 mM). Cells were lysed with radioimmunoprecipitation assay buffer and subjected to immunoprecipitation with anti-Red Fluorescent Protein antibody-coupled agarose (RFP-Trap; Chromotek; rta-10) overnight at 4 °C. After washing, precipitated materials were analyzed by SDS-PAGE and immunoblotting with mouse monoclonal anti-mCherry antibody (clone 8C5.5; horseradish peroxidase–conjugated, BioLegend; 677703), rabbit polyclonal anti-integrin α5 antibody (Proteintech; 10569-1-AP), and rabbit monoclonal anti-integrin β1 antibody (clone EP1041Y; Abcam; ab52971) with secondary horseradish peroxidase–conjugated anti-rabbit IgG (Bio-Rad Laboratories; 170-6515). After reaction with SuperSignal West Pico substrate (Thermo Scientific; 34080) or Dura substrate (Thermo Scientific; 34075), immunoreaction was detected by developing on BIOMAX MR film (Carestream; 870-1302) or by capturing images with Bio-Rad ChemiDoc Touch Imaging System.

### Recombinant TMEM2–integrin binding assay

Recombinant soluble TMEM2 ECD (TMEM2–ECD), tagged at the C terminus with a 6× His epitope, was produced in HEK293 cells, as described previously (25). TMEM2–ECD was bound to ProBond nickel chelating resin (approximately 5 μg protein/20 μl resin; Thermo Fisher) by incubation for 2 h at 4 °C, and the resin was washed with HBSS^++^. Two micrograms of recombinant heterodimer of integrin ECD (α5β1; R&D Systems: 3230-A5-050; αLβ2; R&D Systems: 3868-AV-050) were applied to the TMEM2–ECD–bound and control unbound resin and incubated in HBSS^++^ overnight at 4 °C. After extensive washing, bound materials were eluted by boiling in SDS-PAGE sample buffer, and eluents were analyzed by SDS-PAGE and immunoblotting with rabbit polyclonal anti-integrin α5 (Proteintech; 10569-1-AP), rabbit monoclonal anti-integrin β1 (Abcam; ab52971), rabbit polyclonal anti-integrin β2 (Proteintech; 10544-1-AP), or mouse monoclonal anti-polyhistidine (Sigma; A7058; clone: HIS-1, peroxidase conjugated). Detection of immunoreactive bands was carried out as described as previously.

## Data availability

All data are contained within the article and supporting information.

## Supporting information

This article contains supporting information.

## Conflict of interest

The authors declare that they have no conflicts of interest with the content of this article.
