# Supplementary material for: The cell surface hyaluronidase TMEM2 regulates cell adhesion and migration via degradation of hyaluronan at focal adhesion sites
Source: J Biol Chem. 2021 Feb 26;296:100481. doi: 10.1016/j.jbc.2021.100481 (PMC8042168; doi:10.1016/j.jbc.2021.100481)
Supplement: Supplemental Figure S1 [file mmc1.pdf]

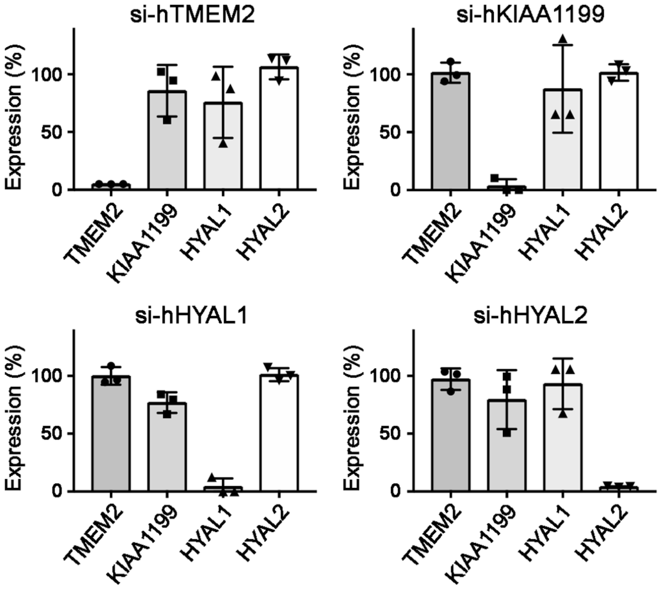

Fig. S1: Effects of siRNAs on hyaluronidase expression in U2OS cells. U2OS cells were treated with siRNAs to TMEM2 (*si-hTMEM2*), KIAA1199 (*si-hKIAA1199*), HYAL1 (*si-hHYAL1*), or HYAL2 (*si-hHYAL2*) for 3 days, and total RNA was isolated and analyzed by TaqMan qPCR. See Experimental Procedures for the TaqMan primer/FAM-conjugated probe sets used in these assays. Hyaluronidase mRNA was normalized to GAPDH mRNA. Expression of change in siRNA-treated cells to control cells is calculated using ΔΔCt method. Data represent mean ± SD from 3 biological replicates.
